# The Long-Term Adaptations of a Combined Swimming and Aquatic Therapy Intervention in an Adult Person with High-Functioning Autism (Asperger’s Syndrome): A Case Study

**DOI:** 10.3390/healthcare11222986

**Published:** 2023-11-19

**Authors:** Maria Koumenidou, Mariana C. Kotzamanidou, Vassilios Panoutsakopoulos, Panagiotis Siaperas, Victoria Misailidou, George A. Tsalis

**Affiliations:** 1 Faculty of Health Sciences, Metropolitan College of Thessaloniki, 546 24 Thessaloniki, Greece, vmisahlidou@mitropolitiko.edu.gr (V.M.); 2 Institute of Occupational Science & Rehabilitation, Metropolitan College, 151 25 Athens, Greece; psiaperas@mitropolitiko.edu.gr; 3 Biomechanics Laboratory, School of Physical Education and Sport Science at Thessaloniki, Aristotle University of Thessaloniki, 541 24 Thessaloniki, Greece; bpanouts@phed.auth.gr; 4 Occupational Therapy Department, Metropolitan College, 151 25 Athens, Greece; 5 School of Physical Education and Sports Science at Serres, Aristotle University of Thessaloniki, 541 24 Thessaloniki, Greece; tsalisg@phed-sr.auth.gr

**Keywords:** autism spectrum disorder, pervasive developmental disorders, therapeutic exercise, aquatic environment, Eurofit fitness test battery, gross mobility, fine mobility, muscle strength, muscle power

## Abstract

Individuals with High-Functioning Autism present impairments in communication, social interaction, and motor development. A low level of motor skills, namely difficulties in gross and fine mobility, and in motor control, discourage individuals with High-Functioning Autism from being involved in physical activities, resulting in fewer opportunities for social interaction. There is not much evidence available about the effects of regular swimming exercise and/or aquatic therapy on health promotion in adults with High-Functioning Autism. An adult male (22 yrs) diagnosed with High-Functioning Autism participated in a combined 6-month swimming and aquatic therapy program (two sessions/week, 60 min each). The pre- and post-intervention assessments consisted of physical fitness, balance, functional ability, and psychomotor tests. The post-intervention assessments showed improvements in the standing long jump (+100%), hand grip force (+71.7%), bend arm hang test (+123.1%), and the physiological parameters in the 6 min walk test (+10.2%). On the opposite, decrements in the sit-up (−12%) and sit-and-reach test (−6.3%) were observed. It was noted that the participant frequently lost interest and focus quickly, resulting in the abandonment of the exercise. Conclusively, there is a great need for further research on this topic examining a larger adult population.

## 1. Introduction

Today, professionals and the wider community discuss the autism spectrum, where on one side of the spectrum are people with autism who have additional intellectual disabilities and on the other are people with the features of autism but without intellectual disabilities and usually average or high performance on intelligence tests. People on this side of the spectrum today are diagnosed as High-Functioning Autism or, in the past, Asperger’s syndrome. The current paper is focused on this end of the autism spectrum. Autism spectrum disorder (ASD) is a neurodevelopmental disorder with a worldwide prevalence estimated at 0.6% [1]. The common characteristics that individuals with ASD present are difficulties in social interaction, difficulties in verbal and non-verbal communication, repetitive behaviors, and irritability [2]. In addition to behavioral difficulties, individuals on the autism spectrum may have lower, compared to average people, movement performance skills and reduced levels of physical activity [3].

According to the Diagnostic and Statistical Manual (DSM-V) of the American Psychiatric Association [4], the same diagnostic category of the autism spectrum includes individuals who present milder symptoms and have an average or higher than average Intelligence Quotient (IQ). The diagnosis of these individuals is either High-Functioning Autism (HFA) or, in the past, what used to be called Asperger’s syndrome (AS) [5].

People diagnosed with HFA are verbal and do not have significant delays in speech, cognitive development, self-care skills development, adaptive behavior, or any intellectual disability, unlike many autistic people [6]. In addition, individuals with HFA present difficulties in carrying out activities of daily living (ADLs), managing their finances and household, using public transportation, and making a good use of their leisure time [7]. In addition, quite often they have difficulties in efficiently performing motor tasks [8] that require gross and fine mobility. Furthermore, they have reduced core strength [9] and balance [10] due to proprioceptive deficits [11] and visuomotor skills [12,13]. It is suggested that motor impairment is related to severity in the AS [14]. Low levels of performance in motor skills, including clumsiness and lack of efficient motor coordination [15], result in reduced participation in physical activity, poor physical condition, and, therefore, exposure of their health to risk [12]. Thus, it is recommended for people diagnosed with HFA to include physical activity in their habits to improve their health and physical condition, while simultaneously preventing chronic diseases [13].

An appropriate type of exercise for individuals diagnosed with HFA is aquatic therapy and swimming [12,13]. The aquatic environment has been used for rehabilitation purposes for over 100 years and is suggested for the treatment of people with musculoskeletal [16] and neuromuscular [17] diseases. At the same time, it achieves improved physical functions and many psychological benefits [18], such as greater self-confidence, self-esteem, and a sense of accomplishment. In recent years, aquatic therapy has been considered a good choice for people with ASD [19]. The physical properties of the aquatic environment offer a relatively stable somatosensory regulation during the activity in the water and limit the arousal levels [19]. Research suggests that movement in the water favors the acquisition of motor skills and allows the body to practice important skills without many gravitation restrictions. Therefore, people on the autism spectrum can practice and improve movement performance with the complete freedom offered by weightlessness. The findings of a systematic review examining the effects of aquatic activities on physical fitness and aquatic skills in children with ASD indicated that aquatic intervention programs of at least 10 weeks in duration can effectively improve aquatic skills, but it is not clear if physical fitness was enhanced [20]. In another study, significant benefits in both gross motor skills and social behavior were revealed after the implementation of a 12-week aquatic intervention program [21]. Furthermore, researchers recommend the use of music and external verbal stimuli during aquatic exercise [22]. 

A recent review suggested that aquatic interventions improved individuals with intellectual disabilities in endurance and strength, but no clear results were found for balance [23]. Nevertheless, aquatic therapy is suggested to be included in recreation therapy programs as part of adult day services for individuals with ASD [24]. Past research suggests that swimming interventions applied to young adults with ASD for a period of 12 weeks usually result in the improvement of social skills [25]. However, a recent study concluded that the implementation of an aquatic therapy program on a young adult (21 years old) with ASD resulted in fewer repetitive movements and stress reactions, as well as enhanced communication skills [26]. Based on this finding, it is of interest to examine the long-term adaptations in the physical fitness and of swimming and aquatic therapy in adults with HFA. The aim of the present case study was to investigate the effect of a 6-month combined aquatic therapy and swimming intervention applied to a young adult individual with HFA. Selected tests were applied to evaluate endurance, strength, explosive power, balance, flexibility, and performance in psychomotor skills.

## 2. Materials and Methods

### 2.1. Participant

A 22-year-old young adult male diagnosed with HFA (AS) was examined. Sub-signified scoliosis was diagnosed with the convex to the right in the middle of the thoraco-lumbar spine, accompanied by increased lordosis and normal range of the intervertebral spaces. The participant had not received physiotherapeutic intervention at all. He was treated by a psychologist and from 2–12 years old he had speech therapy and occupational therapy. His body awareness and color cognition were good, but he had a problem with laterality.

The selection was made since, to the best of our knowledge, no studies on the effect of aquatic therapy and/or swimming exercise programs on postural control and the physical condition of individuals with a profile common to the participant were found in the literature. Signed consent was obtained by the participant and his carers. The research was conducted according to the guidelines of the Declaration of Helsinki and was approved by the Institutional Review Board of the Metropolitan College of Thessaloniki (2195/12 December 2022).

### 2.2. Intervention

The 6-month intervention took place in a private pool (length: 25 m, depth: 1.7 m, water temperature: 27–28 °C) with a frequency of 2 times a week, except in cases where for personal reasons the session was cancelled. The participant was supervised by an independent therapist during the pool sessions.

Each session was divided into six 10 min segments. In the first 10 min segment, for warm-up, the participant held a swimming board with two hands and swam freely with free-style leg kicks, wearing fins. In the second 10 min segment, the participant held the swimming board with one hand while the other was brought to the unilateral thigh. During the inhalation, he was asked to turn the thoracic and cervical spine to the side and on the exhalation to put the head in the water, while the lower limbs continued to perform free-style leg kicks wearing fins. In the third 10 min segment, the participant held the swimming board with both arms outstretched from a supine position and the lower limbs freely performed free-style leg kicks wearing fins. In the fourth 10 min segment, the participant held the swimming board with one hand, while the other arm performed the backstroke stroke, to gain mobility in the shoulder zone. In the fifth 10 min segment, the participant performed front crawl swimming wearing fins in a faster pace than the previous segment. In the sixth 10 min segment, the participant had the swimming board in his arms from a supine position to relax his back. The closing session comprised backstroke as well as free-style swimming (2 strokes at the early stage and, after 3 months of training, 3 strokes) and was applied for 200 m. Adapted backstroke activating only arms or legs was also included.

For the main part, the supine position was mainly applied to gain head-to-spine alignment, full body muscle elongation, and strengthening. Emphasis was given to the propulsion of the body transfer in the supine position, activating the arms from adduction (0° in the horizontal plane) to 180° abduction without leg kicks. This was progressively developed to the propulsion of the body in the supine position from adduction 0° at the horizontal plane to 180° abduction with backstroke leg kicks. For the activation of the arms, from the supine position, the arms were bent and hung, while the legs kept to the backstroke leg kicks. Backstroke kicking legs with flotation aid stabilization of the lumbar part of the spine, with no arm stroke, was also applied. For the improvement of the lateral flexion of the thoraco-lumbar spine, the right and left lateral flexion with no trunk rotation was emphasized. The stability of the whole body was obtained by the use of flotation aids such as neck collar, waist belt, and ankle cuffs.

The combined aquatic therapy and swimming intervention program was applied twice a week from December 2022 to June 2023. The exercises of the program were the same within the months of the intervention, but the number of repetitions and the break time between the exercises progressively changed. It is noted that every month the intensity of the aquatic workout varied because it was dependent on the participant’s motivation. The main focus of the program was for the participant to stay calm and motivated. Behavioral regression was a state for the participant that delayed the progression and the change of the session training volume. The intensity for each exercise was amplified by increasing resistance using aquatic exercise tools, such as aqua hand paddles, aqua dumbbells, ankle cuffs, and sports fins. The total distance for each exercise was 250 m (i.e., 10 repetitions in the full length of the pool). When the exercise was unilateral, in the first 25 m the participant used one side and at the next 25 m of the return he used the other side.

### 2.3. Testing Procedure

After an initial pre-intervention evaluation (PRE), the intervention was commenced. The re-evaluation (POST) was conducted after the completion of the 6-month intervention program with the same tests, which were conducted in a randomized order.

#### 2.3.1. Anthropometric Characteristics 

Initially, the anthropometric data were obtained. The height (cm) was measured with a wall-mounted stadiometer (Seca 220, Seca Deutschland, Hamburg, Germany) while the examinee stood barefooted with his legs closed, knees fully extended, and with his back to a wall. The body mass (in kg) was measured with a typical electronic scale, while the examinee was asked to remove as much clothing as possible. The body mass index (BMI) was calculated as body mass/body height^2^. The skinfold thicknesses of the right biceps brachii, right triceps brachii, right subscapular, and right suprailiac were also assessed using a skinfold caliper (SlimGuide, Pro-Health Product Ltd., Guangzhou, China). The total body fat (BF) was estimated according to Durnin and Womersley [27] and was expressed as a percentage of body mass.

#### 2.3.2. Balance

Balance was assessed using the Romberg balance tests in random order. The balance tests were conducted with open eyes in bipedal and then in monopodal support with right and left foot in support, and then with closed eyes in bipedal and monopodal support [28]. These tests were found to have good reliability and validity according to previous research [29]. The sensorimotor coordination, i.e., hand–eye coordination, was also evaluated in an upright position with arms open at 90° abduction and then with the eyes closed [28]. An Epsan Selecta stopwatch (Epsan, Lelystad, The Netherlands) was used to measure the scores in the balance test.

#### 2.3.3. Six Minutes Walking Test

Functional capacity was assessed using the 6 min walking test. On an indoor 30 m route with the turning points marked with cones, the participant was instructed to walk back and forth at the fastest pace covering as much distance as possible within 6 min [30]. This test is widely considered an easy, cheap, reliable, and valid test for an adult population with intellectual disabilities, but familiarity is required to obtain reliable values [31].

Immediately afterwards, the participant rated his perceived effort using the Rate of Perceived Exertion scale, namely the self-assessment of effort during the exercise, the shortness of breath, and the perceived fatigue during physical exercise, using the Borg scale [32], since the Borg scale is a valid and reliable tool in a variety of different conditions and population groups. The Epsan Selecta stopwatch (Epsan, Lelystad, The Netherlands) was used to measure the time duration of the test. A Seca 201 (Seca GmbH & Co., Hamburg, Germany) tape measure was used to measure the distance where the test was terminated. Before and after the test, a finger pulse oximeter (OxyoPro, Meditech, Athens, Greece) measured the oxygen saturation. Finally, the heart rate was monitored with a Polar Pacer device and constantly recorded using the Polar Flow 6.15.0 software (Polar Electro Oy, Kempele, Finland).

#### 2.3.4. Eurofit Test Battery

The physical fitness level was evaluated with the Eurofit Battery Test. Research by Tsigilis, Douda, and Tokmakidis [33] showed high reliability and validity in a typical age-matched population, while sufficient reliability was confirmed when executed by people with disabilities [34]. It took 35–40 min to perform and very simple equipment was used [35]. The following tests were selected for the evaluation:

- Sit-and-Reach Flexibility test: the participant sat with the knees extended (the researcher also helped the accomplishment of the full extension), separated at the width of the pelvis, and stacked with the soles to the wall. From this position, the participant tried to reach his toes. The examiner measured the distance between the upper and lower limbs in centimeters [36].
- Plate Tapping test: this was used to assess the limb–eye coordination, i.e., the sensorimotor ability. Two discs with a diameter of 20 cm are placed on a table with their centers 60 cm apart. In the middle of this distance, a rectangular cardboard is placed. The participant rested his non-preferred hand on the rectangle and with the hand to be examined made small tapping movements (right and left) from one disk to another as quickly as possible for 25 complete cycles [37]. The Epsan Selecta stopwatch (Epsan, Lelystad, The Netherlands) was used to measure the time duration of the test.
- Standing Long Jump test: the explosive strength of the legs was evaluated. The participant stood behind a line and was asked to jump forward as far as he could. The maximum horizontal distance achieved from the take-off line to the landing point was measured in cm [38]. The Seca 201 (Seca GmbH & Co., Hamburg, Germany) tape measure was used to measure the distance.
- Sit-up test of Abdominal Strength and Muscular Endurance: this was used to evaluate the strength and endurance of the abdominals. The participant lay in a supine position with the knees bent at 90°. The legs were immobilized with the grip of an assistant. The number of sit-ups the participant performed within 30 s was documented [37].
- Bend Arm Hang test: this test assessed the functional capacity and muscular endurance of the upper limbs [26]. The participant hung from a yoke with hands at shoulder width, palms facing forward and elbows bent, so that his chin and gaze stayed above the bar. The test was terminated when the participant’s eyes went under the bar [39].

#### 2.3.5. Hand Grip Strength Test

The maximum isometric hand grip strength was evaluated using the K-grip v2 dynamometer (Kinvent Biomecanique, Montpellier, France) which was found to be valid and reliable [40]. The participant sat on a chair with a straight back, feet on the ground, shoulders relaxed, elbows bent 90°, and the forearm in a neutral position. The participant was evaluated using his preferred hand at first by applying his maximum isometric effort for 5 s with 10 s rest. Then, the other arm was evaluated. The results were extracted using the Kinvent Physio Excellence Application (Kinvent Biomecanique, Montpellier, France). The applied force was recorded in kg [38].

#### 2.3.6. Respiratory Function

Initially, the duration and the quality of inhalation and exhalation were checked with simple observation. Aerobic capacity was then tested by asking the participant to inflate a light and then a hard balloon [41].

#### 2.3.7. Somatognosia

Somatognosia was evaluated by asking the participant to show the different parts of the body initially on an image printed on paper and then the anatomical parts of his body in front of a mirror [42].

#### 2.3.8. Body Awareness

Body Awareness was evaluated by asking the examinee to paint with the color indicated in the corresponding box on the evaluation sheet [42].

#### 2.3.9. Laterality

Laterality was assessed by placing vertically an adhesive tape starting from the center of the sternum to the pelvis. Then, the participant was asked to show or lift various parts of his body right or left [42].

#### 2.3.10. Lateral Side-Bending Flexibility Test

This test assessed the spinal curve mobility [43]. The participant stood upright against the wall where two parallel lines 15 cm apart at right angles to the wall were drawn. The arms were held straight at the sides of the body. The height of the middle finger on each side of the body was marked on the thigh. The participant slowly bent sideways as far as possible while maintaining contact with his back against the wall. The distance between the start and end position of the middle finger was recorded with the Seca 201 (Seca GmbH & Co., Hamburg, Germany) tape measure.

### 2.4. Statistical Analysis

Except for the 6 min walking test, where one attempt was allowed, all tests were conducted three times and the average value was recorded for the pre- and post-intervention comparison. Descriptive statistics are presented as mean value (M), standard deviation (SD), and the coefficient of variation [CoV = SD × 100/M].

## 3. Results

### 3.1. Anthropometric Parameters

The anthropometric parameters are presented in Table 1. The BMI and BF were slightly reduced in the post-intervention measurement.

### 3.2. Strength Tests

The results of the strength tests are provided in Table 2. Except for the sit-up test, improvements were revealed.

### 3.3. Flexibility Tests

The results of the flexibility tests are depicted in Table 3. With the exception of the lateral bend test on the right side, an improvement was observed.

### 3.4. Romberg Balance Tests

Table 4 presents the results of the balance tests. Non-conclusive results were found concerning all conditions (bilateral vs. unilateral stance, open vs. closed eyes, right vs. left leg).

### 3.5. Aerobic Capacity

The results of the 6 min walking test are presented in Table 5, where improvements are evident.

### 3.6. Psychomotor Tests

The results of the psychomotor tests are depicted in Table 6. In the plate tapping test, mixed results were revealed, as there was improvement for the right but not for the left arm.

## 4. Discussion

The present case study investigated the effect of a 6-month combined aquatic therapy and swimming intervention applied to a young adult individual with HFA on endurance, strength, explosive power, balance, flexibility, and performance in psychomotor skills. 

During the majority of the psychomotor tests, the participant confused the right with the left and the examiner had to indicate which end or side the participant was asked to activate. It is suggested that the cerebral hemisphere organization that applies to laterality tests is different in ASD children [44]. This can be the basis for the lateral difference in the plate tapping tests. There was an 11.3% improvement in the right arm and a 6.5% decrease in the left arm. Despite the above-mentioned interlimb difference, the sensorimotor coordination of the eyes and hands was relatively good. Thus, the aim to improve motor performance by incorporating motor coordination in the arm functions via movement planning [45] was partly achieved. 

There were inconclusive results regarding the balance test. There were lower scores (ranging between −4.1% to −6.7%) in two out of three tests with open eyes, while a large decrement was observed in the monopodal balance test with support on the left leg and closed eyes (−80.5%). On the opposite, an increase of above 60% was revealed for the balance tests with support on the left leg (open eyes) and right leg (closed eyes). According to the scores obtained from the balance tests, low scores compared to balance assessments of the typical population were observed due to various possible reasons. The most common reason is the cerebellum differences in people with autism compared to the typical population [46], with the cerebellum being responsible for sensorimotor coordination hypotonia, dysmetry, increased postural instability, and poor coordination [46]. Also, neuroimaging studies of people with HFA have seen reductions in basal ganglia physiology and volumetric decreases in gray matter. This evidence probably explains the abnormal coordination in the corresponding coordination tests, such as the tapping test. As mentioned earlier, no similar interlimb adaptations were observed between the pre- and post-measurement, while the performance of the right upper limb seemed to lag behind the left. 

During the Romberg tests, intense sway and continuous weight transfer were observed during the two-legged stance. Individuals with HFA were shown to have large motor deficits with difficulties in proprioception and multiarticular coordination during movement [47]. The fact that balance appeared to be poorer when the eyes were closed, but without a complaint of vertigo or dizziness, shows that this imbalance was due to the proprioceptive sensory system and not to the visual or vestibular system [48]. Clumsy children are slow to perform a variety of perceptual-motor activities and often exhibit stiff gait patterns, odd posture, poor handling skills, and deficits in visuomotor coordination [38]. However, the researchers seem to conclude that this clumsiness manifests itself more socially than in motor skills, especially in challenging environments [49]. In many cases, the measurements of the motor performance of individuals with autism were completed with difficulty as they lacked motivation or deliberately fell due to boredom. This behavior is not unusual, since people on the autism spectrum find it difficult to start and complete a specific activity [5].

Fitness markers in people with autism show relatively low levels of cardiovascular endurance, lower limb flexibility, and upper body muscle strength and endurance compared to the typical population [12]. During the pre-measurement, 540 m were covered in the 6 min walking test, while for typically developed individuals the average distance is 698 ± 96 m [50]. In the post-measurement, there was an obvious improvement with the participant covering a total of 720 m (+33.3%). The odd fact was that oxygen saturation was low even at rest, compared to a normal value of 99%. This is typical, as research showed that when the parasympathetic nervous system is activated, autistic individuals have significantly increased pulse and decreased oxygen saturation, which is probably related to excessive response and overstimulation of the brain [51]. On the Borg scale, the participant scored 6/10 on both measures, meaning that the level of exercise was severe with a lot of sweating and muscle strain and relatively high heart rate and partial shortness of breath. 

Physical conditioning is affected by activity levels. Participation in physical activity for autistic individuals can be difficult due to poor motor skills, lack of motivation, and limited opportunities for physical activity [9]. Another factor that can affect their physical performance is sensory hypersensitivity, which is related to noise and touch. This affects the energy levels during an activity, as well as participants’ responses to issues related to pain, discomfort, energy, and fatigue [52]. In this case study, the positive effects on the cardiorespiratory system of the participant were evident, after the encouragement of his carers for systematic exercise, despite the limiting factors mentioned above that could have prevented him from following a more physically active lifestyle.

On the other hand, there were no improvements or remarkable alterations in the hamstring flexibility (−6.4%) and lumbar spine mobility (−0.7% to −5.4%) tests, respectively. Therefore, the lack of an effect of the intervention on stiffness could be associated with the intense kyphoscoliosis of the participant, something that is also confirmed by past research [53]. Referring to the relative literature to confirm this information, it was found that there is a complete lack of research on stiffness in a population with AS. Thus, it is of interest to examine this factor in the future. It is also interesting to examine the factors associated with the fact that no lateral flexion improvements were observed despite the considerable increase in the front flexion (+17.6%).

The tests of muscle strength and endurance in the upper limbs and torso showed the following: in the Bend Arm Hang test, the participant simply held the handles without being able to pull over, which proved his reduced muscular strength and endurance in the upper limbs. However, in the post-measurement, there was a mentionable improvement (+123.1%) compared to the pre-measurement regarding the time duration in which he was held hanging which may indicate an improvement in the muscular endurance of his upper limb and his back muscles. In the Hand Grip, the participant scored a considerable increase (+86.0%) of the muscle strength in the right hand, while the left had an obvious lower increase (+57.4%). This can also be attributed to the lateral differences that are evident in individuals with ASD. However, as can be seen from the detailed analysis of the data, in the first two attempts, the participant performed lower than average and only in the third attempt in the left hand he achieved a better result. Finally, concerning the number of sit-ups in both the pre- and post-measurement, they were in reasonable agreement with the scores of typically developed individuals [54]. 

Poor motor skills in individuals with AS are related to significantly more abnormalities in the head than trunk posture compared to healthy populations [15]. In this particular case, where there was also significant kyphoscoliosis; the therapist who supervised the participant in the pool reported a limited range of motion in the shoulder joints, a large chest extension, and a large immersion in water, even though fins and a swimming board were used. Nevertheless, over the course of the months, there was a positive development in terms of rotation in the trunk, better coordination, and slightly better mobility in the arms despite the minimal mobility of the shoulder blades.

This study is not free of limitations. Recording data regarding the movement kinematics and the internal load during the intervention program could add context to the evaluation of the development of the participant’s physical fitness. In addition, the use of stabilographic measurements could have provided further insight into the biomechanical parameters of stance control. Another limitation is the lack of monitoring of the differences in the cognitive factors provoked by the intervention. The cognitive potential is a factor of interest when examining the effectiveness of aquatic therapy in individuals with ASD [55], with research providing evidence about the enhancement of the cognitive abilities [56]. Finally, a follow-up measurement session was not conducted due to the summer holidays.

However, it is important to mention that, due to AS, the participant was often not in the mood to engage and very easily gave up exercise or was removed, lost motivation, showed signs of boredom, or, at the end of the hour-long session, he used to stop abruptly without having completed the corresponding measurement. Problems in motor coordination may affect the emotional state of individuals with HFA as it happens with people with Developmental Coordination Disorder who are described by their carers as isolated, easily frustrated, introverted, and socially immature. Also, they have poor self-esteem and self-perception concerning physical activities. They avoid the playground and have less positive interaction with their classmates [57]. This behavior suggests that they are at risk of jeopardizing their participation in social activities and, combined with their reduced performance in motor skills, they have limited participation in community sports teams or free play time with other children, compared to typically developing children, resulting in lower levels of physical activity [58]. 

In the present case study, the aquatic therapy intervention assisted the participant in gaining relatively good coordination of the limbs. Regarding the flexibility and mobility of the trunk, there was no improvement and, in many cases, there was a deterioration of scoring in the above-mentioned tests. The results of the tests related to muscle strength, such as the Bend Arm Hang, Hand Grip, and sit-ups, showed positive signs after the intervention. Finally, the parameters associated with endurance were enhanced as well. In conclusion, the measures of balance, jumping ability, and functional capacity before and after the intervention revealed a positive development, given the fact that usually the aquatic therapy interventions have the lowest weekly frequency compared to other interventions applied to individuals with ASD [25].

## 5. Conclusions

Based on all the above, it follows that a combined swimming and aquatic therapy intervention for a person on the autism spectrum offers significant benefits in physical fitness. The intervention aided the participant in becoming familiar with a variety of stimuli, in socializing, in creating a beneficial habit, and in improving his cardio-respiratory system and functional capacity. In general, there is a great need for further research on what therapeutic swimming can offer to people with autism, especially in high-functioning adults, with the urge to examine larger samples for a more detailed comprehension.

## Figures and Tables

**Table 1:** Pre- and post-intervention results for the anthropometric parameters.

Parameters	PRE	POST
Body height (m)	1.665 (0.0) [0.0]	1.665 (0.0) [0.0]
Body mass (kg)	56.3 (0.0) [0.0]	55.8 (0.0) [0.0]
Body mass index (kg/m^2^)	20.3 (0.0) [0.0]	20.1 (0.0) [0.0]
Biceps skinfold thickness (mm)	4.0 (0.0) [0.0]	4.0 (0.0) [0.0]
Triceps skinfold thickness (mm)	13.0 (0.0) [0.0]	13.0 (0.0) [0.0]
Subscapular skinfold thickness (mm)	10.0 (0.0) [0.0]	9.7 (0.6) [6.0]
Suprailiac skinfold thickness (mm)	8.0 (0.0) [0.0]	9.0 (0.0) [0.0]
Body fat (%)	14.6 (0.0) [0.0]	14.5 (0.2) [1.4]

NOTE: values represent mean value (M), standard deviation (SD), and the coefficient of variation (CoV).

**Table 2:** Pre- and post-intervention results for the strength tests.

Test	PRE	POST
Standing long jump (cm)	66 (0.0) [0.0]	132 (5.3) [4.0]
Hand grip force—left (kg)	4.7 (1.0) [21.1]	7.4 (2.0) [26.6]
Hand grip force—right (kg)	5.0 (1.6) [32.1]	9.3 (2.9) [32.1]
Bend arm hand (s)	3.9 (1.7) [42.5]	8.7 (3.7) [42.5]
Sit-ups (#)	16.7 (3.5) [21.1]	14.7 (2.5) [42.5]

NOTE: values represent mean value (M), standard deviation (SD), and the coefficient of variation (CoV).

**Table 3:** Pre- and post-intervention results for the flexibility tests.

Tests	PRE	POST
Sit-and-reach (cm)	32.0 (2.0) [6.3]	34.0 (0.0) [0.0]
Forward bend (cm)	33.0 (2.7) [8.0]	38.8 (0.3) [0.8]
Lateral bend—right (cm)	55.3 (3.8) [6.8]	52.3 (4.0) [7.7]
Lateral bend—left (cm)	51.0 (6.1) [11.9]	54.7 (2.5) [4.6]

NOTE: values represent mean value (M), standard deviation (SD), and the coefficient of variation (CoV).

**Table 4:** Pre- and post-intervention results for the balance tests.

Tests	PRE	POST
Bilateral stance—open eyes (s)	30.0 (0.0) [0.0]	28.0 (7.2) [25.8]
Bilateral stance—eyes closed (s)	30.0 (0.0) [0.0]	30.0 (0.0) [0.0]
Unilateral stance—right leg—open eyes (s)	24.3 (9.8) [40.3]	23.3 (0.3) [0.8]
Unilateral stance—right leg—eyes closed (s)	18.7 (12.0) [64.5]	30.9 (6.1) [19.8]
Unilateral stance—left leg—open eyes (s)	7.4 (2.3) [31.2]	12.1 (9.2) [75.8]
Unilateral stance—left leg—eyes closed (s)	8.7 (8.4) [96.6]	1.7 (0.7) [40.4]

NOTE: values represent mean value (M), standard deviation (SD), and the coefficient of variation (CoV).

**Table 5:** Pre- and post-intervention results obtained at the 6 min walking test.

Parameter	PRE	POST
Heart rate—pre-test (bpm)	80	78
Heart rate—post-test (bpm)	112	95
Distance covered (m)	540	720
Oxygen saturation—pre-test (%)	98	95
Oxygen saturation—post-test (%)	94	97
Balloon inflation (YES/NO)	YES	YES

**Table 6:** Pre- and post-intervention results obtained for the psychomotor tests.

Test	PRE	POST
Plate tapping test—left arm (#)	26.2 (1.7) [6.6]	24.5 (0.4) [1.8]
Plate tapping test—right arm (#)	24.7 (0.3) [1.3]	27.5 (2.5) [9.2]
Somatognosis (YES/NO)	YES	YES
Body Awareness (YES/NO)	YES	YES
Laterality (YES/NO)	NO	NO

NOTE: values represent mean value (M), standard deviation (SD), and the coefficient of variation (CoV).

## Data Availability

The data that were acquired and analyzed in the present study are available from the corresponding author upon reasonable request.
